# Recent Progress in Two-Dimensional Magnetic Materials

**DOI:** 10.3390/nano14211759

**Published:** 2024-11-01

**Authors:** Guangchao Shi, Nan Huang, Jingyuan Qiao, Xuewen Zhang, Fulong Hu, Hanwei Hu, Xinyu Zhang, Jingzhi Shang

**Affiliations:** 1 Institute of Flexible Electronics (IFE), Northwestern Polytechnical University (NPU), 127 West Youyi Road, Xi’an 710072, China; iamguangchao@mail.nwpu.edu.cn (G.S.); dqiaojy@mail.nwpu.edu.cn (J.Q.); zhang_xuewen@mail.nwpu.edu.cn (X.Z.); hufulong@mail.nwpu.edu.cn (F.H.); hanwei.hu@mail.nwpu.edu.cn (H.H.); xinyu_zhang@mail.nwpu.edu.cn (X.Z.); 2 Fifth Research Institute, China Electronics Technology Group Corporation, 524 Zhongshan East Road, Nanjing 210016, China

**Keywords:** 2D magnet, spin order, ferromagnetic, antiferromagnetic, spintronics

## Abstract

The giant magnetoresistance effect in two-dimensional (2D) magnetic materials has sparked substantial interest in various fields; including sensing; data storage; electronics; and spintronics. Their unique 2D layered structures allow for the manifestation of distinctive physical properties and precise performance regulation under different conditions. In this review, we present an overview of this rapidly developing research area. Firstly, these 2D magnetic materials are catalogued according to magnetic coupling types. Then, several vital effects in 2D magnets are highlighted together with theoretical investigation, such as magnetic circular dichroism, magneto-optical Kerr effect, and anomalous Hall effect. After that, we forecast the potential applications of 2D magnetic materials for spintronic devices. Lastly, research advances in the attracting magnons, skyrmions and other spin textures in 2D magnets are discussed.

## 1. Introduction

Magnetism behaves as a natural reaction to a magnetic field, and the macroscopic magnetism of a substance mainly arises from internal electron spin. In general, magnetic materials can be divided into two categories based on the spin order: magnetically ordered materials, such as ferromagnetic (FM) and antiferromagnetic (AFM) materials, and magnetic disordered materials, including paramagnetic and diamagnetic materials. These materials can be used in sensors, Hall devices, and magnetic information recording devices. Despite significant progress, the fundamental science of magnetic materials remains incompletely understood, leaving room for further advancements. As the quality of the emerging magnetic materials and devices continues to be improved, there is potential for significant breakthroughs to revolutionize the field soon [1].

With the emergence of graphene, advances in studying materials with varying dimensions have unveiled intriguing possibilities. Specifically, materials may exhibit completely disparate properties with dimensional variation. For example, the previous work by De Jong and Miedema outlined the critical impact of dimensionality on magnetic behaviors [2], using simplified model systems to elucidate the fundamental interactions within different dimensional magnetic lattices. Additionally, Nikolaos et al. proved spin transport and spin precession phenomenon in monolayer graphene at room temperature and completed spintronic transport at micron-level distances [3]. Furthermore, Han et al. investigated the tunneling effect in doped graphene, which made progress in studying spin relaxation [4]. After introducing 2D magnetism into graphene by nitrogen doping, a saturation magnetization of up to 1.09 emu/g was obtained [5], which has extensive prospects in spintronic devices.

After discovering graphene and thoroughly exploring its properties, most attention began diverting from three-dimensional (3D) to low-dimensional materials based on a relatively complete theoretical understanding of 3D magnetic materials. The Mermin–Wagner theorem is an essential theoretical tool used to understand the properties of 2D magnetic materials, in which the stability of long-range magnetic order is poor in isotropic 2D magnetic materials due to the effect of thermal fluctuation [6]. However, the Mermin–Wagner theorem cannot be used to determine the long-range FM order in the system at low temperatures because the broken rotational symmetry of long-range bond orientation involves all atoms in the system [7]. Therefore, investigating magnetic materials requires a thorough understanding of the Mermin–Wagner theorem and consideration of other factors, such as lattice structure and electron interactions, to understand the magnetic behaviors comprehensively.

**Figure 1:**
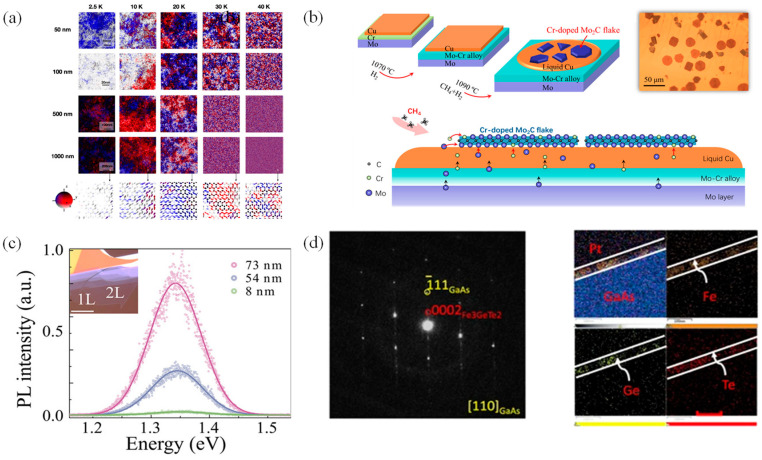
(**a**) Magnetic spin image of a honeycomb lattice at variable temperature and variable system size obtained by Monte Carlo step, the scale bar is displayed on the first image of each row (reproduced with permission from [8], Springer Nature, 2022). (**b**) Schematic illustration of the growth process for the Cr-doped α-Mo_2_C and the optical image of Cr-doped α-Mo_2_C flakes (reproduced with permission from [15], American Chemical Society, 2021). (**c**) The PL spectral intensity of the two-dimensional CrBr_3_ varies with thickness (reproduced with permission from [20], American Chemical Society, 2019). (**d**) SAED images taken from the interface area of the Fe_3_GeTe_2_ thin film grown on (111) GaAs and EDS mapping of Fe, Ge and Te atoms with a scale bar of 100 nm (reproduced with permission from [31], Springer Nature, 2017).

The researchers utilized different physical models such as the honeycomb lattice model, the hexagonal model (Figure 1a) [8], and the amorphous solid model separately [9,10,11] and obtained different types of 2D magnetic materials through surface engineering such as point defects and doping (Figure 1b) [12,13,14,15,16,17,18,19]. Two-dimensional magnetic materials are limited to the plane due to carrier migration and heat diffusion, making them have many properties not available in 3D materials. For example, the atomic utilization is increased by the higher specific surface area of 2D magnetic materials, and carrier motion is limited to the in-plane further to increase ion transport rates at the microscopic level. The thinner structure provides good flexibility and transparency at the microscopic level. Due to the unique nature of crystal structures, different 2D magnetic materials may exhibit anisotropy, resulting in different electrical or optical properties. In addition, 2D magnetic materials are appealing owing to the tunneling resistance effect and easily adjustable magnetism. So far, the mechanical exfoliation method [20,21,22], molecular beam epitaxy [23,24,25], chemical vapor deposition (CVD) method [26,27,28], and other processes have been used to manufacture 2D magnetic materials. As shown in Figure 1c, the photoluminescence (PL) spectral intensity of 2D CrBr_3_ significantly depends on the thickness of the material. As the thickness of the CrBr_3_ changes, the emission intensity observed in the PL spectrum varies accordingly [20], highlighting the importance of controlling and precisely measuring the thickness of CrBr_3_ for light-emitting-related applications. Figure 1d illustrates the SEAD image of wafer-scale 2D FM Fe_3_GeTe_2_ thin films grown using molecular beam epitaxy. Experimentally, 2D magnetic materials can be obtained by exfoliating layered magnetic crystals into thin layers. In 2017, interesting 2D magnetic behaviors were discovered in monolayer CrI_3_ and bilayer Cr_2_Ge_2_Te_6_ [29,30,31]. However, the poor conductivity and low magnetic transition temperature of these materials present obstacles to their practical device applications at room temperature. Deng et al. discovered a new 2D intrinsic magnetic material, Fe_3_GeTe_2_, which increases the magnetic transition temperature of the sample to room temperature through lithium-ion intercalation [32], and the device fabrication technique may be extended to other 2D magnetic materials. A recent comprehensive review by Jiang et al. delves into these advancements, particularly emphasizing the discovery of 2D magnetic ordering in materials like CrI_3_ and Fe_3_GeTe_2_ [33], which covers the magnetic exchange interactions in these materials, including direct, superexchange, and double exchange mechanisms, and discusses the importance of these interactions in influencing magnetic properties. Since then, 2D magnetic materials have attracted increasing attention in the 2D material family [34,35]. Table 1 presents a comprehensive overview of 2D magnetic materials and classifies their types.

## 2. Physical Properties of 2D Magnetic Materials

### 2.1. Magnetic Circular Dichroism

**Figure 2:**
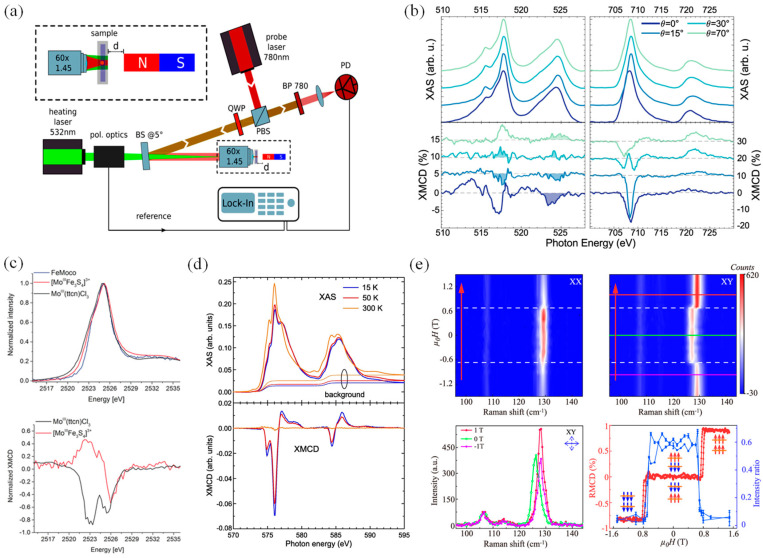
(**a**) The structure of photothermal circular dichroism microscope developed from magnetic circular dichroism (reproduced with permission from [77], American Chemical Society, 2022). (**b**) Background-corrected white line spectra taken at θ = 0°, θ = 15°, θ = 30°, and θ = 70° for the V_L2,3_-edges and the Fe_L2,3_-edges of Fe_1_V_1_/Cu (001) and the XMCD (reproduced with permission from [83], Springer Nature, 2021). (**c**) Normalized Mo L3-edge XAS and XMCD spectra of Mo^III^ (ttcn) Cl_3_ (black) and [Mo^III^Fe_3_S_4_]^3+^ (reproduced with permission from [90], American Chemical Society, 2019). (**d**) Temperature-dependent XAS and XMCD measurements of CrI_3_ at the Cr edges in a field of 2 T (reproduced with permission from [89], Elsevier, 2018). (**e**) Magnetic field dependence of the polarized Raman spectra for bilayer CrI_3_ (reproduced with permission from [91], American Chemical Society, 2020).

Magnetic circular dichroism (MCD) is often associated with the phenomenon of electrons’ transition to different excited states under the external magnetic field. In addition, these different excited states also absorb left or right circularly polarized light, exhibiting the characteristic of circular dichroism [75,76]. The difference in applications between MCD and circular dichroism mainly depends on the physical mechanism and the specific selected wavelength range. MCD can observe electronic transitions that are difficult to observe in ordinary light absorption spectra. The structure of the photothermal circular dichroism microscope is shown in Figure 2a, which has been developed based on the principles of MCD [77]. The photothermal circular dichroism microscope allows for enhanced imaging and analysis capabilities by utilizing the advancements in MCD techniques [77].

At present, MCD has been widely used in the field of biology [78,79,80]. With the emergence of 2D magnetic materials, MCD and its associated effects are anticipated to be utilized on such 2D magnets. X-ray magnetic circular dichroism (XMCD) can be used to measure circularly polarized photons to obtain a dichroic intensity difference proportional to the atomic magnetic moment, whose magnitude is affected by factors such as the degree of photon polarization, the angle between the photon angular momentum, the magnetic moment, and the expected value of the magnetic moment. Such XMCD effect has been employed to fabricate magnetic microscopes with spatial resolution better than 15 nm and spectral energies ranging from 250 eV to 1.8 keV [81]. Furthermore, it can be integrated with spatial-resolved instruments to investigate the magnetic properties of elements on or even below the surface of materials [82], including the distribution of spin and orbital angular momentum, magnetic moment, and magnetic coupling strength [75].

The magnetic moment direction can be determined by observing the positive and negative XMCD peaks of sub-monolayer (<0.03 ML) Fe and V adatoms, revealing the degree of AFM coupling between the magnetic moments of these two elements; further examination of the relationship between the XMCD peak and the incidence angle shows that the coupling mechanism of the magnetic moment of Fe and V is more intricate than initially assumed (Figure 2b) [83]. By integrating X-ray resonance magnetic reflectance (XRMR) data to analyze alterations in the spatial distribution of magnetic moments and XMCD data, researchers discovered a high degree of data consistency after scrutinizing average magnetic moment changes alongside magnetic proximity effects, highlighting the advantage of XRMR in the spin field of magnetic material [84,85].

The XMCD results of specific elements in 30 nm FeNi thin films prove that the magnetic anisotropy is affected by the magnetic moment of the Fe spin–orbit [86]. The low-temperature magnetic order of 2D CrSiTe_3_ is mainly derived from the anisotropy of Cr ions. Further measurements of X-ray absorption spectroscopy (XAS) and XMCD show the superexchange between Cr atoms playing a significant role in the low-dimensional magnetic sequence arrangement [87,88,89,90], which unveils the origin of such a low-dimensional magnetic order (Figure 2c,d). Moreover, colloidal few-layer CrI_3_ was measured by MCD, in which the bilayer CrI_3_ device could achieve reversible gate regulation compared with the monolayer counterpart [13]. Correlating the reflected MCD with Raman spectral images revealed the presence of hysteresis loops, providing robust evidence that the applied magnetic field caused spin flipping in CrI_3_ (Figure 2e) [91]. The transition from AFM to FM in the interlayer of atomically thin CrI_3_ under the adjusted pressure was observed using MCD and electron tunneling measurements, enabling the regulation of the interlayer magnetic ground state [92]. In CrI_3_ field-effect devices, the magnetic order has been characterized by MCD, indicating that magnetic sequence flipping can be achieved by controlling the external electric field [93]. These studies highlight the significant potential of 2D van der Waals (vdW) magnetic materials in the field of spin transistors or spin field-effect transistors.

### 2.2. Magneto-Optical Kerr Effect

The magneto-optical Kerr effect (MOKE) and MCD both can be triggered by a polarized light source. However, MCD generally reflects the differential absorption of circularly polarized light through a magnetic material in which the amplitude difference is affected by the material’s magnetism. By contrast, MOKE frequently occurs when the polarized light is reflected from the magnetic material. According to the magnetization angle and the medium plane, the relationship between the incident surface can be further divided into longitudinal, polar, and transverse Kerr effects. As a result, the magnetic properties can be measured separately by adjusting the magneto-optical Kerr angle.

**Figure 3:**
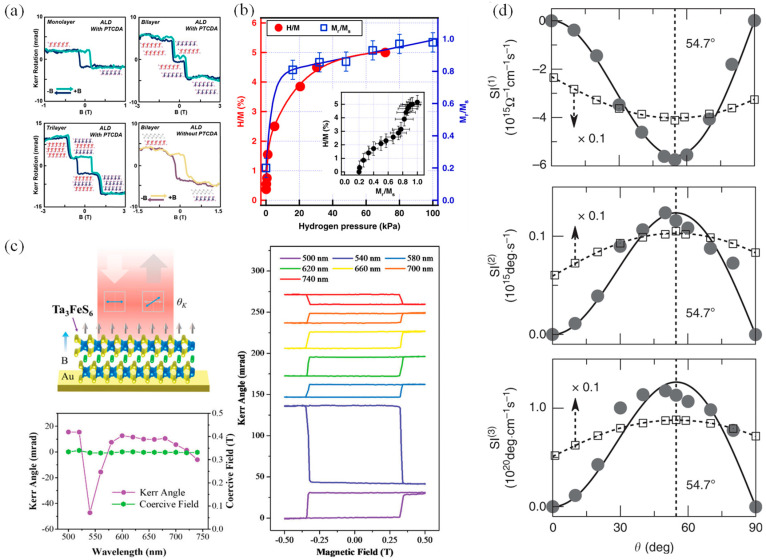
(**a**) MOKE of ALD-encapsulated CrI_3_ with and without an organic buffer layer (reproduced with permission from [96], American Chemical Society, 2021). (**b**) The correlation between hydrogen concentration and residual magnetic saturation ratio is determined by MOKE (reproduced with permission from [97], American Chemical Society, 2021). (**c**) T_c_ of Ta_3_FeS_6_ nanosheet can be determined by MOKE (reproduced with permission from [101], American Association for the Advancement of Science, 2020). (**d**) Magnetic sequence image of the spectrum of the real part of the non-diagonal magnetic conductibility in the strained γ-Fe_0.5_Mn_0.5_ system, as well as the spectra of the Kerr angle and Faraday rotation angle (reproduced with permission from [102], Springer Nature, 2020).

The MOKE exhibits a robust capability in detecting low-dimensional materials [94]. Its non-invasive nature ensures it does not disrupt the magnetic order by destroying the material layer structure [95]. Thus, MOKE is an ideal technique for detecting 2D magnetic materials. For instance, MOKE has been effectively utilized in studying bilayer CrI_3_ and perylene tetracarboxylic dianhydride encapsulated by alumina film (Figure 3a) [96], as well as hydrogen diffusion in 50 nm cobalt-palladium films (Figure 3b) [97], in which the long-range magnetic coupling is affected by the reversed magnetization. Additionally, 2D CrTe flakes obtained by CVD were analyzed using MOKE and MCD, where highly ordered spin directions and hard FM properties were related to strong vertical anisotropy [98]. Furthermore, the properties of magnetic films can be determined by selecting the appropriate substrate and optical wavelength with the assistance of MOKE [99,100]. The 2D Ta_3_FeS_6_ exhibited intrinsic long-range FM order and excellent stability under the atmosphere, in which the Curie temperature (T_c_) of 80 K was identified for few-layer Ta_3_FeS_6_ using MOKE, as illustrated in Figure 3c [101].

In addition to FM materials, MOKE can be expected in AFM materials, such as strained γ-Fe_0.5_Mn_0.5_ (Figure 3d) and CrBr_3_/CrI_3_ bilayers [102,103]. Due to the shared symmetry requirements between the anomalous Hall effect and MOKE in materials, MOKE may also be observed in antiferromagnets exhibiting the anomalous Hall effect; for example, large zero-field Kerr rotation was observed in an AFM metal Mn_3_Sn at room temperature [104]. In 2D CrI_3_, the polar Kerr effect shows significant variation with the applied magnetic field in Faraday geometry, making it suitable for detecting the hysteresis effect of the Kerr rotation under the external magnetic field, in which monolayer CrI_3_ was identified as an Ising ferromagnet with the vertical spin orientation [29]. Besides, MOKE was utilized to detect the magnetoelectric effect of gate regulation in bilayer CrI_3_ gating devices [105], in which the switching between AFM and FM states was controlled by the applied voltage. Moreover, theoretical studies have predicted that the bilayer structure formed by combining the monolayers of CrI_3_ and CrBr_3_ exhibits intralayer FM and interlayer AFM with distinct MOKE responses, suggesting potential applications in memory devices [103].

### 2.3. Anomalous Hall Effect

The Hall effect is typically observed in a conducting material when a magnetic field is applied perpendicular to the current direction, resulting in a transverse potential difference (i.e., Hall voltage) across the material. This is known as the ordinary Hall effect, where the applied magnetic field plays a crucial role. By contrast, with the non-zero internal magnetization, the anomalous Hall effect can be expected without an external magnetic field, often observed in FM materials. Current consensus attributes the internal cause of the anomalous Hall effect to the breakdown of spatial inversion symmetry, leading to the generation of orbital magnetism by non-zero Berry curvature; meanwhile, external factors, such as the scattering of conductive electrons and impurities within the crystal lattice, also contribute to this phenomenon [106]. Traditionally, the anomalous Hall effect was thought to occur primarily in solids and independent of AFM order. However, recent studies have demonstrated its presence even in some antiferromagnets with minimal magnetization; for instance, adjusting the direction of the moment in the Mn triangular spin structure of Mn_3_Sn can induce the anomalous Hall effect [107]. Additionally, the optical anomalous Hall effect has been observed in monolayer graphene, as evidenced by the presence of ultrafast abnormal Hall currents in optical guide switch detection devices [108]. The anomalous Hall effect holds significant promise in the realm of microscopy, offering avenues for studying dissipation-free transport and quantum topological phenomena. Back in the last century, Klitzing et al. found that Hall resistance in field-effect transistors changed gradually but did not display a linear relationship with magnetic field intensity [109], i.e., the quantum Hall effect; the mechanism of the quantum Hall effect is that the edge electrons move directionally under the magnetic field without scattering, so the corresponding longitudinal resistance becomes zero when the Hall resistance reaches a plateau. The low-dissipation properties of the quantum Hall effect are excellent [110], but the cost of realization in the macroscopic field is unacceptable. Such phenomena have inspired researchers to explore the quantum anomalous Hall effect through extensive experimental and theoretical investigations [111].

**Figure 4:**
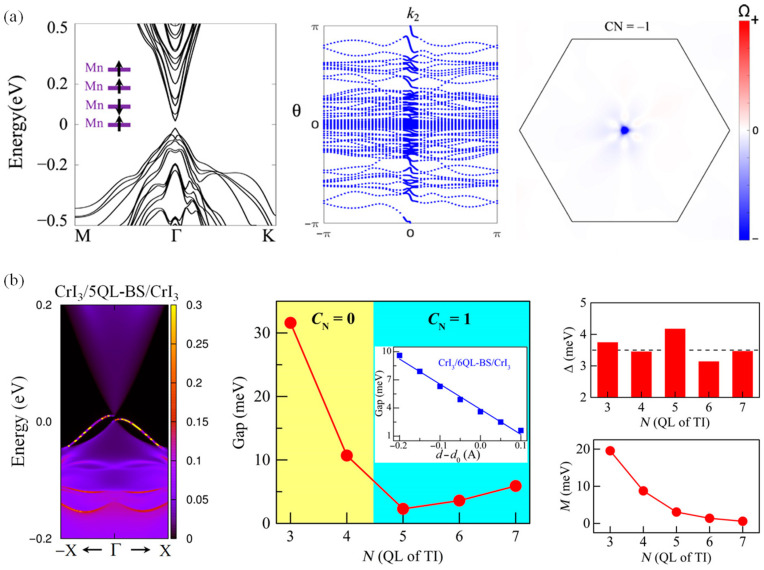
(**a**) Special magnetic order and the associated anomalous Hall effect of 2D MnBi_2_Te_4_ in the interface (reproduced with permission from [119], American Association for the Advancement of Science, 2020). (**b**) Topological properties of CrI_3_/BS/CrI_3_ (reproduced with permission from [118], American Association for the Advancement of Science, 2019).

After the discovery of the quantum Hall effect, researchers began investigating the possibility of a quantum anomalous Hall effect and sought to verify this hypothesis. The quantum anomalous Hall effect is characterized by the anomalous Hall conductance reaching a critical value and forming a plateau at zero magnetic field, with the chemical potential positioned within the energy gap opened at the Dirac point of an FM topological insulator. Under these conditions, the longitudinal conductance is expected to be zero. Initially, it was believed that several conditions must be satisfied in order to observe the quantum anomalous Hall effect: the material’s band structure must possess topological properties and support a conductive one-dimensional edge state; there must be a long-range FM order to induce an anomalous Hall effect; and the material must be insulating so that only the one-dimensional edge states contribute to conduction. Hence, magnetically doped topological insulators were considered to be promising candidates for achieving the quantum anomalous Hall effect. Subsequently, theoretical calculations and data analysis suggested that breaking time inversion symmetry without a magnetic field could lead to a non-zero topology number, thereby inducing the Hall effect [112,113,114,115,116]. However, obtaining evidence to prove the existence of the quantum anomalous Hall effect remained challenging. In 2013, Xue’s team first discovered the quantum anomalous Hall effect in experiments [117], and the research in related fields began to develop rapidly [11]. Density functional calculations indicate that Bi_2_Se_3_ and monolayer FM CrI_3_ can produce considerable spin splitting at the Dirac point of the topological surface state [118]. Additionally, by coupling MnBi_2_Te_4_ of the AFM magnetic sequence with 2D CrI_3_ to form a heterojunction, the disorder of magnetic moment and the influence of magnetic domain on the surface magnetic mechanism in MnBi_2_Te_4_ is theoretically solved, and the anomalous Hall effect is regulated without an applied magnetic field (Figure 4a) [115,119]. Moreover, stacking heavy element atoms (such as Bi) on 2D CrI_3_ can create a natural splitting surface and a large band gap, resulting in a quantum Hall effect, and the band structure of this system can be further adjusted by altering the spin direction (Figure 4b) [118,120].

Expanding on the exploration initiated by the discovery of the quantum Hall effect, researchers have delved into the properties of 2D CrI_3_ doped films grown via molecular beam epitaxy, where these films exhibit residual anomalous Hall resistance at low temperatures, along with a notable contrast between transverse and longitudinal resistance [121]. This discrepancy suggests the presence of a quantum anomalous Hall effect, with its manifestation influenced by the inherent anisotropy of the crystal structure [121]. These findings contribute to our understanding of quantum phenomena and lay a foundation for future device applications.

### 2.4. Tunnel Magnetoresistance Effect

**Figure 5:**
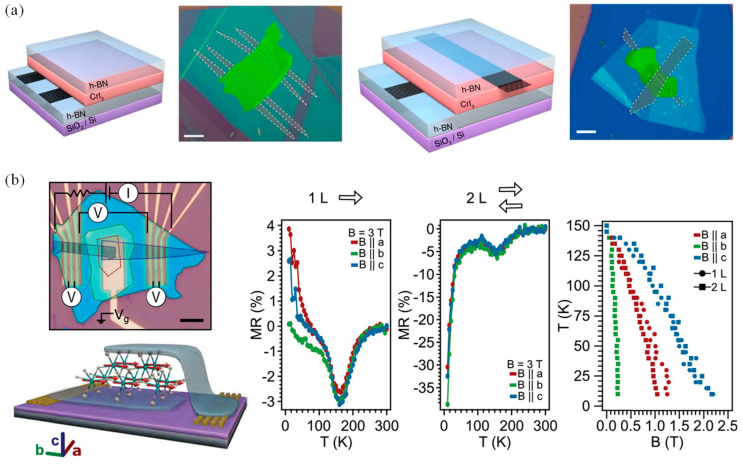
(**a**) Semiconducting characteristics of CrI_3_ FET with tunnel magnetoresistance effect (reproduced with permission from [127], Springer Nature, 2018). (**b**) Magnetic characteristics of 2D CrSBr vdW heterojunction (reproduced with permission from [128], John Wiley and Sons, 2022).

In magnetic tunnel junctions (MTJs), tunnel magnetoresistance (TMR) arises from spin-related tunneling effects, and these junctions typically consist of a non-uniform magnetic system comprising magnetic layers separated by an insulating layer [122]. Switching control is achieved by adjusting the magnetic alignment of the electrodes, often facilitated by the giant magnetoresistance effect. The thin, nonmagnetic, and non-conductive layer between the two FM layers enables the tunnel magnetoresistance effect, which allows for precise adjustment of the magnetic order of one FM layer without affecting the other. This characteristic offers promising applications in sensing and storage due to its large magnetoresistance effect and high magnetic field sensitivity [123,124,125,126]. Figure 5a presents the semiconducting characteristics of a 2D CrI_3_ field-effect transistor (FET) with TMR effect. However, the fabrication of multilayer MTJs using traditional methods on 3D materials can introduce numerous defective structures at the interfaces of different materials, thereby compromising the performance of the MTJs [127]. Recent efforts have been made to address this challenge by using 2D magnetic materials combined via vdW forces to create magnetic tunnel structures with easy regulation and high response sensitivity (Figure 5b) [128]. Moreover, theoretical calculations involving MXene applied to MTJs have shown promising results, with similar lattice parameters and adjustable physical and chemical properties, in which a maximum tunneling reluctance ratio of 6.95 × 10^6^% was obtained in vdW MTJs with 1T-MoS_2_ as an electrode and 2H-MoS_2_ as a tunnel barrier [129]. Furthermore, spin valve devices based on 2D Fe_3_GeTe_2_ have exhibited high tunneling reluctance ratios, indicating their potential for applications in spintronics and magnetic tunneling devices [130].

Nowadays, 2D magnetic material CrI_3_ has emerged as a key candidate for MTJs due to its tunable magnetic properties, high spin polarizability, ease of preparation, and stable performance. Theoretically, by modulating the FM–AFM transition in 2D CrI_3_, significant changes in TMR can be achieved, which have reported values of up to 3000% TMR in Cu/CrI_3_/Cu structures [131], and an astonishing 19,000% spin-filtering TMR in graphene/CrI_3_/graphene MTJs [132]. The potential barrier level in the FM insulator is influenced by the electron spin direction, thereby manipulating the AFM–FM transition. Experimental evidence suggests the tunnel barrier thickness can be reduced to less than 10 nm while significantly enhancing the conductance per unit area [133]. Despite variations in the bias voltage, 2D-CrI_3_-based MTJs maintain high spin-filtering performance [134]. Furthermore, owing to intralayer FM and interlayer AFM properties of CrI_3_ flakes, their MTJs exhibit pronounced parity-layer effects and spin-filtering phenomena, leading to layer-dependent tunneling resistance correlations [135,136,137]. The MTJs composed of 2D CrI_3_ can achieve a TMR ratio of 1.48 × 10^14^, and the TMR of the vdW MTJs is much larger than that of the transverse MTJs [138]. The magnetic anisotropy of 2D CrI_3_ can be manipulated by chemical adsorption, substitution doping, and photoexcitation, offering avenues for developing advanced spintronic devices with enhanced efficiencies and operational speeds [139].

### 2.5. Magnetic Proximity Effect

**Figure 6:**
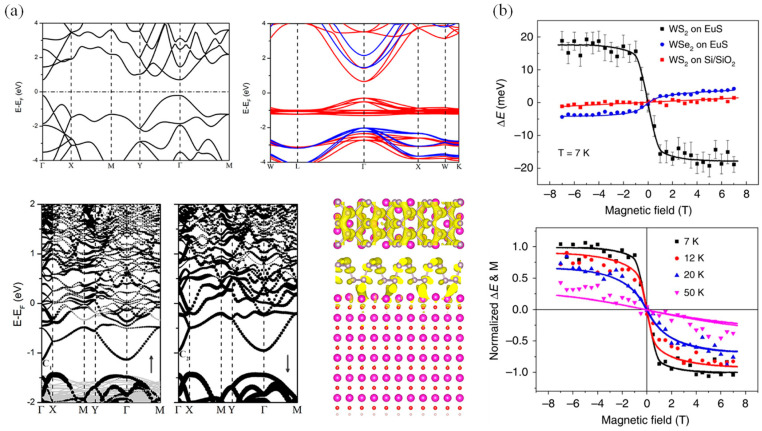
(**a**) The energy region of the band structure of phosphorene can be impacted by the proximity effect effectively. Red and blue lines represent the spin up and down bands, while gray spheres represent the P atoms, red spheres the O atoms, and white spheres the H atoms (reproduced with permission from [142], American Chemical Society, 2022). (**b**) In 2D WS_2_, the magnetic proximity effect leads to Valley Zeeman splitting (reproduced with permission from [147], Springer Nature, 2019).

The magnetic proximity effect often describes the phenomena of spin polarization occurring at the interfaces between nonmagnetic and FM or AFM materials due to the exchange interactions. In 1969, Pb-Pd-(Fe or Cr), Pb-Ni, and Pb-Mo-Cr multilayer structures were prepared at 77 K by inspiratory evaporation and sputtering technology, in which the magnetic penetration of the spin polarization of magnetic metals into the nonmagnetic materials was found [140]. In recent years, with the emergence of 2D materials and improved growth techniques, the magnetic proximity effect has played an essential role in spintronic structures and nanodevices (Figure 6a) [141,142,143,144], in which 2D SnO/CrN heterostructures, monolayer BP/EuO, ultrathin-Fe-overlayer/Bi_2−x_Mn_x_Te_3_, and 1.7 nm Pt layer/CoFe_2_O_4_ were proposed or employed.

The orbital hybridization can be adjusted by applying an electric field, allowing for control over the band structure through the magnetic proximity effect at the interface [145]. According to the first-principles calculations, the band structure of the vdW heterojunction of MoS_2_/CrI_3_ and its manipulation via a vertical electric field have been studied, where an electric field of 0.115 V^−1^ can generate a considerable valley split of about 19.60 meV [146]. Figure 6b shows that the magnetic proximity effect induces valley Zeeman splitting in 2D WS_2_ and WSe_2_ on the magnetic EuS substrate [147]. PL and reflected MCD have been used to detect the WSe_2_–CrI_3_ heterojunction, confirming that it can achieve short-range proximity effects due to the vdW stacking [147]. Additionally, the mechanical exfoliation method for creating heterojunctions can lead to uneven forces on the material, resulting in an uneven distribution of magnetic domains. For example, strain can induce changes in the magnetic sequence of the CrI_3_ layer, further affecting the magnetic proximity effect [148,149], where the analysis of Zeeman separation energy and Zeeman and applied magnetic fields reveals the magnetic proximity effect. In the case of graphene/CrBr_3_ heterojunctions, the magnetic proximity effect strongly depends on the orbital hybridization at the interface [150], which is generated by spin-dependent interlayer coupling, consistent with the observation of the spin Hall effect signal at zero field in graphene/CrBr_3_ [151].

### 2.6. Second-Harmonic Generation

Second-harmonic generation (SHG) generally refers to the process of annihilating two optical photons with a fundamental frequency of ω simultaneously, producing a second-harmonic photon with a frequency of 2ω. Notably, there are no conventional energy-level-involved transitions during this process. Two conditions for SHG are often required: the material has a non-centrosymmetric structure, and the phase-matching relationship is satisfied [152].

**Figure 7:**
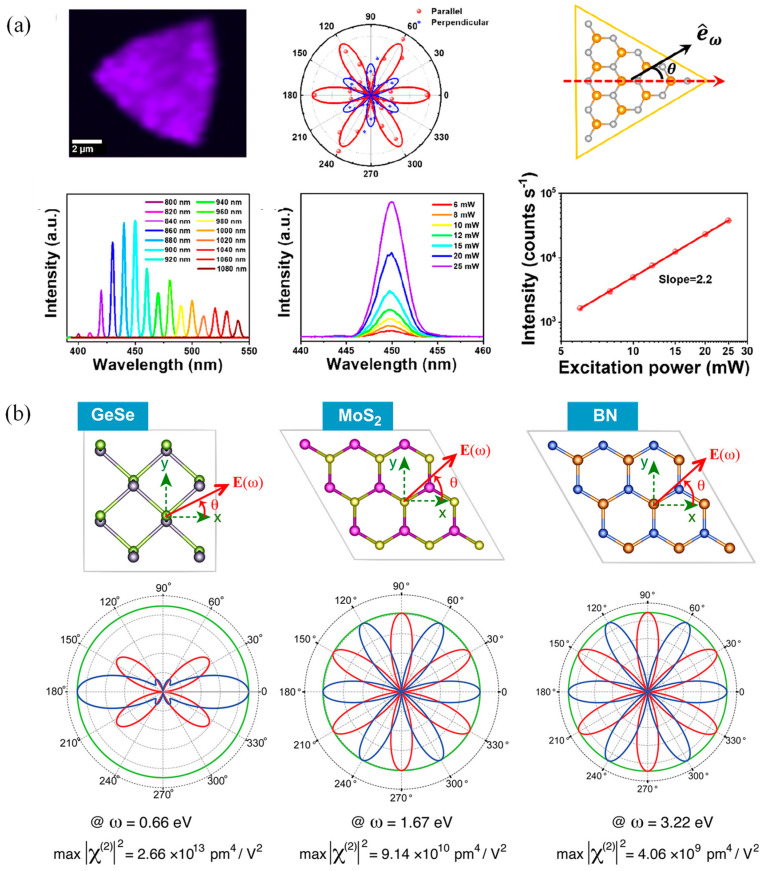
(**a**) SHG characterization of 2D VSe_2_ (reproduced with permission from [154], American Chemical Society, 2022). (**b**) Predication of SHG graphics in several materials. The red and blue lines are |χ_∥_^(2)^(θ)|^2^ and |χ_⊥_^(2)^(θ)|^2^, respectively. (reproduced with permission from [159], American Chemical Society, 2017).

At the microscopic level, SHG can arise from the broken inversion symmetry, crystal structure, and spin configuration, such as ferroelectricity and AFM. Near-field SHG offers superior resolution performance for characterizing microstructural information and enhancing SHG detection capabilities; for example, the magnetic anisotropy and broken inversion symmetry of monolayer VSe_2_ in the H-phase have been uncovered by SHG measurements (Figure 7a) [153,154,155]. Moreover, the strong excitonic effect in atomic-thin transition metal chalcogenides can enhance SHG and improve detection quality, in which nanoscale variations in the stacking order between layers in bilayer WSe_2_ have been observed by using near-field SHG imaging approaches [156]. Moreover, 2D halide metal perovskites exhibit strongly adjustable SHG characteristics under polarized light [157], while plasma combined with monolayer transition metal dichalcogenides (TMDCs) enables continuous tunability of output wavelength [158]. The predicted SHG spectra in GeSe, MoS_2_, and BN are depicted in Figure 7b [159], which illustrates the anticipated SHG profiles across different materials, providing insights into their nonlinear optical properties under varying experimental conditions.

Importantly, magnetic phase transitions in 2D materials can be characterized by SHG. For example, the SHG have demonstrated that the T_c_ of monolayer CrSBr is approximately 146 K [52], in which the SHG intensities of even-numbered layers are significantly higher than the ones of odd-numbered layers due to the parity effect in few-layer CrSBr. Electric dipole SHG mechanisms play a role in even-numbered layers rather than odd-numbered layers, and the Néel temperature (T_N_) exhibits an increasing trend with the decreasing layer number of CrSBr, possibly due to intermediate phases between the FM and AFM phases [52]. As seen above, SHG can effectively detect the crystal structure of 2D magnetic materials, magnetic phase transitions, and other related properties. These findings lay a solid foundation for further research in low-dimensional magnetism.

### 2.7. Theoretical Calculations of 2D Magnetic Materials

Till now, numerous experiments have identified various types of 2D magnetic materials. However, relying solely on experiments and empirical knowledge to discover such materials has its limitations. Theoretically, the first-principles calculations based on density functional theory (DFT) are a reliable approach to analyze and predict potential 2D magnetic materials, opening up the opportunities for ideal magnetic material selection.

In general, DFT employs the electron density of particles, significantly reducing the operational parameters and thereby enhancing computational efficiency, which is particularly suitable for theoretical systems where high accuracy is not required. Although the DFT initially proposed by Hohenberg et al. has been widely used, it was primarily suited for studying ground-state properties, and its applicability to excited states remained uncertain [160,161]. However, recent advancements have extended the application of DFT to excited states [162]. In particular, DFT utilizes electron density to depict the interactions among multiple electrons, yielding local magnetic moments consistent with the strongly correlated AFM eigenstates of the Heisenberg Hamiltonian, indicating that DFT offers a description of quantum mechanical eigenstates in line with theoretical models [107]. In a DFT study, vibrational, electronic, magnetic, and topological properties of a subset of 258 compounds were calculated and explored, identifying 56 FM and AFM systems [163], and the subsequent high-throughput calculations screened 89 magnetic monolayers [164]. According to the DFT and Monte Carlo modeling calculations, bilayer CrI_3_ exhibits a temperature-related significant magnetoelectric response, positioning it as one of the most valuable 2D magnetic materials [165]. Furthermore, external factors can modulate the 2D magnetic properties, which can also be explored by DFT calculations. For examples, oxygen defects in 2D magnetic materials can elevate magnetic anisotropic energy and the Curie temperature in 2D CrI_3_ [166], while compressive and tensile strains alter magnetism direction and magnetization, resulting in changes in T_c_ and specific heat capacity [167,168].

## 3. Spintronics

Spintronic devices often incorporate spin properties into semiconductor devices by utilizing both electron charge and spin as information carriers. The interlayer interaction, magnetic proximity effect, and other microscopic characteristics of 2D magnetic materials can significantly enhance the performance and responsiveness of spintronic devices. However, the magnetic response is influenced by various factors, including inhomogeneous chemical composition, crystal structure, surface morphology, interface properties, and magnetic domain structure. For instance, 2D CrSBr exhibits strain-adjustable magnetism, and the introduction of strain along different crystal directions leads to distinct effects on the magnetic domains [169]. In addition, the T_c_ of 2D FM CrSBr can be effectively elevated up to 175 K by applying strain, and triaxial magnetic anisotropy can be introduced, holding crucial implications for practical spintronic devices [170]. The FM–AFM transition in twisted bilayer CrI_3_ can be controlled by external current, which enables the coexistence of the FM phase and the AFM phase under uniform distribution conditions, offering the potential for the realization of more complex magnetic ground states in future spintronic devices [171].

**Figure 8:**
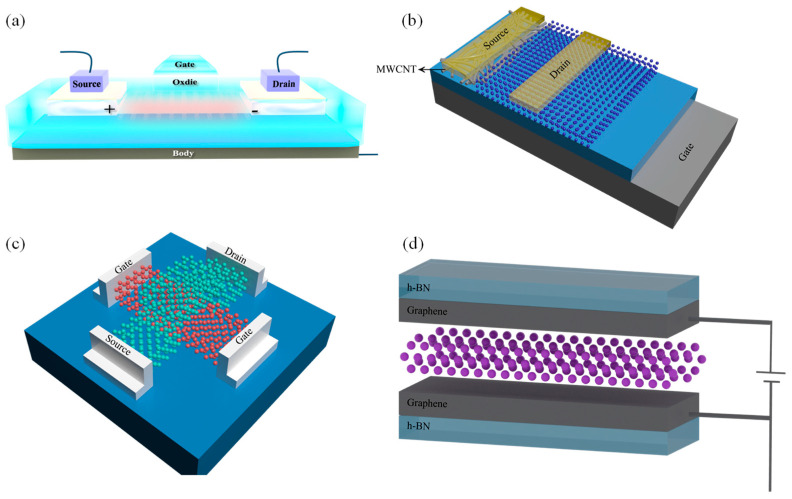
Illustration of the FET and MTJ architectures. (**a**) Illustration of the MOSFET architecture. (**b**) The structure of vertical tunnel field-effect transistor. (**c**) The junction field-effect transistors with the ambipolar channel. (**d**) Magnetic tunnel structure of FM layer/insulating layer/FM layer in h-BN package.

The FET is an essential electronic component that regulates current through electric field effects, which generally requires an appropriate band gap to minimize switching losses, high carrier mobility for responsive regulation, and high thermal conductivity to mitigate damage from device heating. To meet these demands, the metal-oxide-semiconductor field-effect transistors (MOSFETs) are a typical device structure (Figure 8a,c), in which the 2D magnet can be employed as the functional layer. Moreover, complementary metal-oxide-semiconductor circuits, known for their robust interference resistance and fast operation, have been developed based on such device architectures [172]. Nonetheless, with the size shrinkage of devices, the distance between the source and drain electrodes in conventional MOSFETs reduces, possibly causing the tunneling current, and the excessive input voltage may provoke latch-up, which may impede FET functionality. Instead, tunneling FETs (TFETs), as a promising new technology, employ quantum tunneling to address the issue of high energy consumption. The schematic structure of vertical TFETs is illustrated in Figure 8b,d, in which the current can flow from the source to the channel under an appropriate gate voltage by band-to-band tunneling. Although their performance often lags behind MOSFETs at higher voltages, TFETs exhibit the capability to operate at significantly lower voltages and substantially reduce off-state leakage current.

Previously, Radisavljevic et al. demonstrated promising low-power MoS_2_-based transistor applications with mobility rates reaching 200 cm^2^/V·s at room temperature [173], which spurred the investigation of various 2D materials, like MoSe_2_, WS_2_, PrSe_2_, and PtS_2_, for FET applications [174,175,176,177,178,179]. In particular, 2D magnetic materials are promising candidates due to the giant magnetoresistance effect, which allows their resistances to be altered by magnetic field. Applying tensile/compression strain induces lattice deformation in a 2D FM semiconductor CrS_2_, leading to a change in spin mode from spin up to spin down; when the tensile strain exceeds 3%, the material undergoes a transition from semiconductor to semi-metal [180]. Similarly, the magnetic sequence of CrI_3_ can be adjusted by applying current, which results in improved spin-filtering effects, making it suitable for application in spin FETs [181].

Incorporating diverse materials into the source regions of MOSFETs and TFETs has led to the development of hybrid devices, exhibiting improved power dissipation across various drive voltages [182]. Beyond the FETs, organic field-effect transistors (OFETs) composed of π-electron-conjugated systems containing aromatic rings have aroused research attention due to their lightweight nature, excellent flexibility, and ease of large-scale manufacturing. Planar OFETs, despite their widespread use, face limitations in transmission rates due to the width of their conductive channels and are prone to bias-stress instability [183,184]. Conversely, vertical OFETs, with channel lengths defined by the thickness of their layers, offer narrower conductive channels; such architectures facilitate high current densities and operating frequencies at lower voltages and ensure the minimal charge transfer disruption even when the device is mechanically bent [185,186].

## 4. Magnons

Magons represent quasiparticles of collective excited states of the electron spin structure in the lattice, visualized as quantized spin waves in quantum mechanics. These excited states, which are the basis for understanding the magnetic interactions in solids, manifest as wave-like phenomena where spins in a crystalline lattice oscillate around their equilibrium positions. In 1957, Bertram Brockhouse probed the direct measurement of magnons through inelastic neutron scattering in ferrites, providing experimental evidence of their physical properties [187].

Spin waves describe a coordinated wave-like motion of spins in magnetic materials. Unlike magnons, which are quantized disturbances or “particles” of spin waves, the term “spin wave” refers to the collective oscillation itself, which can be described in classical terms without involving quantum mechanics. Magnons emerge when these spin waves are considered under quantum mechanical frameworks, highlighting their particle-like behavior as discrete energy packets of the spin wave [188,189]. The relationship between the magnons and spin waves is similar to the case between photons and electromagnetic waves in an optical field. Magnons are quantized units of spin waves, in which each carries specific energy and momentum determined by its wave vector.

**Figure 9:**
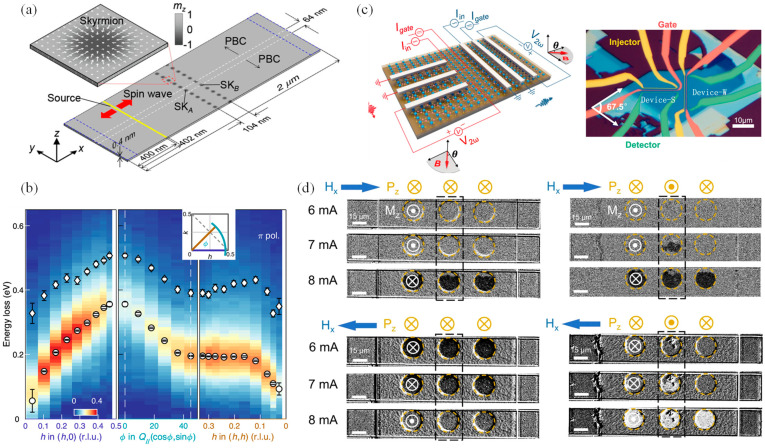
(**a**) Schematic illustration of the sample structure (reproduced with permission from [192], American Physical Society, 2016). (**b**) Magnon and bi-magnon dispersions (reproduced with permission from [203], Springer Nature, 2024). (**c**) Anisotropic magnon transport measurement device (reproduced with permission from [214], Springer Nature, 2023). (**d**) Real-time voltage regulation of magnon torque. Yellow and white (⊙|⊗) symbols indicate the direction of the ferroelectric polarization for BiFeO_3_ and magnetization for PtCo, respectively. (reproduced with permission from [218], Springer Nature, 2024).

Spin waves are collective excitations in spin systems of magnetic materials, manifested as magnetic moment fluctuations of oscillations of specific lattice points within these materials. The theoretical foundation of spin waves includes multiple physical concepts, such as magnetization, magnetic anisotropy, exchange interactions, and dipolar interactions [190]. The spin-wave dispersion and its propagation dynamics are critical for the study of 2D magnetic materials and are essential for understanding magnetic interactions and their applications in spintronics. The dispersion relation of spin waves, i.e., the relationship between their frequency and wave vector, reveals the fundamental dynamics of spin wave propagation [191]. Under the influence of an external magnetic field, spin waves can propagate in specific directions within magnetic materials; their propagation speed and decay characteristics depend on the material’s magnetic anisotropy, exchange interaction strength, and geometric dimensions [191]. Moon et al. introduced a novel method to control spin wave propagation in 2D magnetic devices through an array of skyrmions acting as magnetic antennas, and Figure 9a shows a schematic diagram of the experimental setup [192]. This work achieved precise control of spin wave propagation by altering the skyrmion array configurations and adjusting their position with an asymmetric magnetic field, enabling modification of spin wave refraction and propagation characteristics without physical movement of the structure and effectively guiding spin wave behavior through the topologically stable properties of skyrmions [192]. Moreover, Nikolaev et al. focused on the generation of second-harmonic spin waves in 2D materials, a phenomenon involving the production of new waves at twice the original frequency through the interaction of fundamental spin waves [193]. By controlling the wave vector and frequency of spin waves to ensure phase-matching conditions, they enhanced the efficiency of the second-harmonic generation, advancing the understanding of nonlinear spin wave dynamics and improving the technological potential of magnonics for the design of next-generation magnonic circuits and devices [193]. Furthermore, the excitation of spin waves is the basis for studying the dynamical properties of 2D magnetic materials, typically involving the use of external magnetic fields or microwave fields to excite collective oscillations of magnetic moments in these materials. A common excitation method is using microwave pulses, whose frequency matches with the magnetic resonance frequency of the material, effectively exciting spin waves [194]. Chen et al. explored the magnonic band structure of CrI_3_ using inelastic neutron scattering, analyzing spin wave excitation in the 2D honeycomb lattice of CrI_3_ [195]. The study revealed two distinct FM excitation bands, attributed to significant internal magnetic fields caused by Dzyaloshinskii–Moriya interactions [195], which not only advanced our understanding of magnetic excitations in 2D materials but also highlighted their potential in creating dissipationless spintronic devices.

During the resonant excitation process of spin waves, the amplitude of the spin waves reaches its maximum, and the efficiency of energy transfer is significantly enhanced [196]. The phenomenon of spin wave resonance plays a vital role in the applications of spin waves, especially in improving the precision of spin wave control and energy transmission. Note that such techniques have been proved to be feasible for magnetic crystals of YFeO_3_ to probe collective spin waves [197]. Understanding the dispersion relations, excitation, and resonance mechanisms of spin waves lays the foundation for exploring their broader impact in thermodynamics. The thermodynamic effects of spin waves focus on their interactions with the thermal properties of 2D magnetic materials during propagation and include the generation of thermally excited spin waves, heat transport, and thermomagnetic effects induced by spin waves [198]. After discussing the excitation and propagation properties of spin waves, a further comprehending of the quantum process of spin waves can help to reveal their deeper physics. Especially when the energy of spin waves is quantized, the concept of magnons arises, inherently behaving as oscillators that follow Bose–Einstein statistics [199]. This quantization, usually a result of the intrinsic quantum mechanical nature of electron spins in the lattice, marks a significant aspect of spin wave behavior and applications.

In recent years, many achievements in magnons have been made in experiments. The development of nanofabrication and precision measurement technologies now allows scientists to study magnons at smaller scales and higher temporal resolutions [200,201]. For instance, Bartram et al. employed sophisticated spectroscopic techniques to examine the influence of layer thickness on the magnetic properties of 2D MnBi_2_Te_4_ [202], where they successfully tracked the demagnetization process induced by laser pulses and directly observed the dynamics of spin waves. Wang et al. conducted a study using resonant inelastic X-ray scattering to examine magnons interactions in a 2D Mott insulator [203] and identified both single and bi-magnon dispersions (Figure 9b), which underscores the importance of quantum corrections for precise descriptions of magnon dynamics; furthermore, the study emphasizes the significance of SrCuO_2_’s proximity to a quantum critical point, which may facilitate the discovery of novel magnetic states.

Recent studies [204,205,206,207,208] on magnons in 2D materials have not only highlighted their fundamental dynamics but also demonstrated their unique topological properties. One notable example is the magnon Hall effect, similar to the electronic Hall effect but driven by spin waves rather than charge carriers [204,205]. In 2D materials, this effect leads to a transverse magnon flow without an external magnetic field, attributed to spin–orbit coupling and Dzyaloshinskii–Moriya interactions that disrupt inversion symmetry and introduce topological characteristics to the magnon band structure [206]. Similarly, the topological Hall effect, primarily observed in topological spin textures like skyrmions, arises from spin structure asymmetry [207,208]. This effect results in a topological response as magnons navigate through these textures, creating real-space Berry curvature [207,208]. Crucially, these phenomena are characterized by robust edge states, or topologically protected edge states, that allow magnons to travel along material edges without being affected by scattering or defects, expanding their applicability in spintronics and quantum computing [209,210]. Topologically protected bound magnon states forms a quasiparticle state that remains stable even during external interference or structural defects. Moreover, the spin Seebeck effect is another important phenomenon in 2D magnetic materials, where a spin current is generated under a temperature gradient and carried by magnons [211,212]. This effect, first observed in FM insulator materials [213], has been extensively explored in 2D materials. The interaction of spin-driven currents with magnon Hall and topological Hall effects further influences the path of spin current transmission. The chiral movement of magnons, combined with the stability provided by topological spin structures, enhances the functionality of thermoelectric applications [212]. Qi et al. demonstrated in their study on 2D CrPS_4_ that second-harmonic thermal magnons exhibit significant anisotropic transport, which can be electrically controlled using a gate current, as depicted in Figure 9c, with the anisotropic spin Seebeck effect playing a key role in this tunability [214]. These topological properties enable the sophisticated control and manipulation of magnons in 2D materials. Building upon this, the spin waveguiding effect, in particular, utilizes specific geometric configurations to direct spin wave propagation, essential for the precise control and efficient transmission of information in spintronic devices [215].

Magnons contribute significantly to fundamental physics research and hold promising prospects in information technology and quantum computing. As carriers of information in magnetic materials, magnons are considered a vital system for future information systems due to their low energy consumption and high data-processing speeds [216,217]. In a recent study, researchers have demonstrated reconfigurable logic-in-memory via voltage-controlled magnon torque in thin-film multiferroics (Figure 9d) [218]. Utilizing the interplay between FM and AFM orders, this approach allows precise control of magnon behavior, opening new avenues for low-energy, efficient logic devices [218]. In the field of quantum computing, spin waves are used to couple with superconducting qubits, facilitating efficient quantum information transmission and manipulation [219]. Coherent coupling studies between magnons and superconducting qubits have shown that magnons can be used as carriers in quantum information processing, and such coupling allows the exchange of quantum states between qubits and magnons [219]. The field of magnonics is rapidly advancing the innovation of next-generation information technologies. Control and manipulation of magnons are promising in quantum computing and spintronic devices. As research deepens, magnonics is expected to play an increasingly critical role in areas such as low-power consumption, rapid data transmission, and quantum information processing.

## 5. Domain Movement and Skyrmion

**Figure 10:**
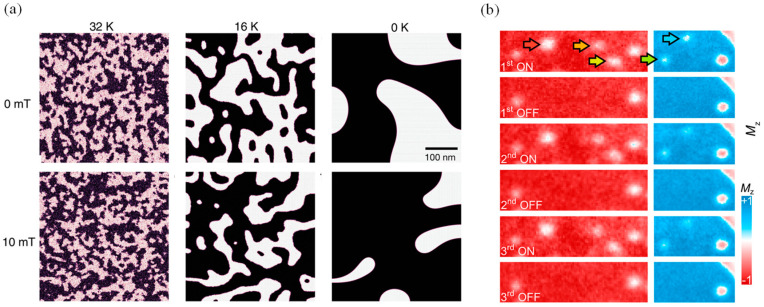
(**a**) Theoretically anticipated magnetic domains in single-layer CrI_3_, different color arrows indicate five skyrmion writing/deleting processes (reproduced with permission from [222], John Wiley and Sons, 2020). (**b**) SPLEEM images show reversible skyrmion writing and deletion over three hydrogen ON/OFF cycles (reproduced with permission from [228], Springer Nature, 2022).

To develop stable spintronic devices, it is crucial to control spin orientation within 2D domains and domain walls. Magnetic materials inherently exhibit magnetic domains and domain walls due to self-induced demagnetization effects. The spin states within these domains and their distributions are vital for the magnetic response, and control of domains is fundamentally essential for understanding 2D spintronics and facilitating the development of related magnetic devices. The 2D magnetic material CrSBr displays paramagnetic behavior above its critical T_c_ due to random spin orientations; the long-range magnetic order above the T_N_ is inhibited by thermal fluctuations, while the short-range magnetic order emerges from intralayer spin coupling below the T_c_, thereby transforming 2D CrSBr into an FM material [220]. Figure 10a presents monolayer CrI_3_ domain walls that exhibit the features of both soft and hard magnets, such as ease of movement, small-area hysteresis, high crystalline anisotropy, and narrow domain walls [221,222]. These walls can induce metastable domains at low temperatures, showing the magnetic characteristics of both Neel and Bloch types [222]. In the twisted double-trilayer CrI_3_, the alteration of interlayer exchange coupling results in the coexistence of AFM and FM domains due to stacking changes between monoclinic and rhombohedral structures, akin to the properties observed in twisted bilayer structures [223]. Such manipulation of magnetic domains through the arrangement of vdW stacking orders suggests that magnetic moiré superlattices could serve as a platform for nanomagnetism exploration.

The quest to develop coherent theories for the intricate domain structures of 2D magnetic materials has been around since the 1960s, when the skyrmion concept was first introduced [224,225] and validated experimentally [226,227]. These nanomagnetic domain structures are notable for their topological protection and the ability to be regulated by various physical factors such as temperature, magnetic fields, and electric fields. Skyrmions are distinguished by their unique chiral spin configuration, low drive current density, and responsiveness to various external conditions (Figure 10b) [228], marking them as fundamental units for future information storage technologies that demand high density, speed, and energy efficiency [229]. In contrast to conventional FM and AFM materials, the arrangement of magnetic moments in a skyrmion vortex confers properties not typically accessible. Notably, skyrmions exhibit localized particle-like characteristics and can possess extremely small magnetic domain sizes, down to 3 nm [230,231]. Pursuing new materials with superior performances is an active research area in magnetoelectronics and is pivotal for advancing the practical deployment of magnetic skyrmions in spintronic devices [232].

The intercalation layers of chromium telluride, denoted as Cr_x_Te_y_, offer a means to modulate magnetic domain distributions, which are exemplified by the independent spin-flip transitions observable in Cr_2_Te_3_ and Cr_5_Te_8tr_ within a 2D Cr_2_Te_3_-Cr_5_Te_8tr_ lateral heterojunction under varied magnetic fields [233]. The phase transition boundary, achieved through continuous interpolation, enables the sequential arrangement of magnetic domains, enhancing the design and functionality of spintronic devices. Furthermore, the field of micromagnetism has facilitated the exploration of innovative domain wall structures in ultra-thin films that exhibit perpendicular anisotropy. Theoretically, the presence of uncompensated Dzyaloshinskii–Moriya interactions between layers can contribute to the formation of these novel structures; for example, such advancements are instrumental in deciphering the mechanisms behind current-induced domain wall motion in films with perpendicular anisotropy, paving the way for further developments in spintronics [234].

## 6. Other Spin Textures

In 2D magnetic materials, a rich variety of magnetic textures exist beyond skyrmions, including bimerons, magnetic vortices, and other skyrmion-like structures with distinct topological features. These textures have been validated through experimental and theoretical analysis.

**Figure 11:**
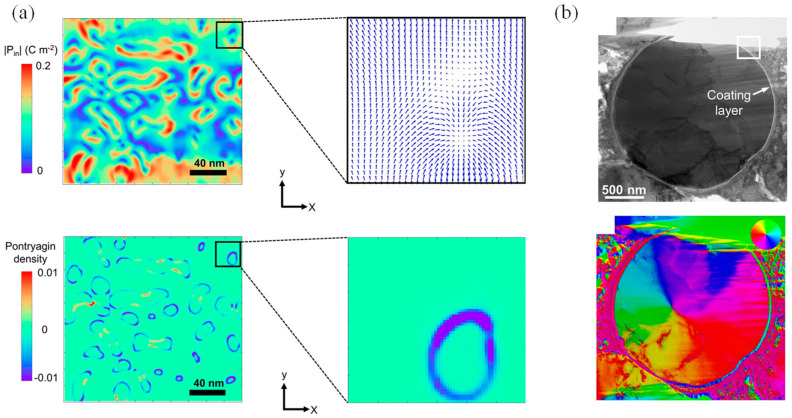
(**a**) A magnified view of the in-plane polarization and Pontryagin density reveals a bimeron structure with a topological charge of −1 (reproduced with permission from [238], Springer Nature, 2023). (**b**) Magnetic vortex structure (reproduced with permission from [242], Springer Nature, 2024).

Bimerons, similar in topology to skyrmions, are characterized by a pair of opposing spin vortices in space [235]. Theoretically, bimerons can be induced in AFM materials through the application of external magnetic fields or electric fields [236,237]. Govinden et al. discovered ferroelectric solitons in BiFeO_3_/SrTiO_3_ superlattices [238], revealing layered structures as small as 3 nm with centric convergent/divergent topological defects; first-principles effective Hamiltonian calculations and phase-field simulations manifest that these structures can be classified as bimerons with ±1 topological charge (Figure 11a). The unique aspect of bimerons lies in their topological protection similar to skyrmions, and their bipolar characteristics, which exhibit distinctive dynamic behaviors under external disturbances [239]. Due to their controllability via external fields, bimerons hold potential applications in spintronics and information storage [240].

Magnetic vortices, commonly found in various magnetic materials, are characterized by complex arrangements of magnetic moments at the vortex core. Experiments have shown that stable vortex structures can be generated in 2D magnetic materials by precisely controlling external magnetic fields or strain [241]. Vortices are significant not only in low-dimensional magnetic systems but also have practical applications in spintronic devices [242,243]. Recent studies highlighted a new type of soft magnetic composite based on magnetic vortex structures [242], in which ultrafine FeSiAl particles were isolated by an Al_2_SiO_5_/SiO_2_/Fe_2_(MoO_4_)_3_ multilayer heterostructure to form a singular magnetic vortex structure with the reduced coercivity and the enhanced compressive strength (Figure 11b).

Additionally, other complex topological structures like topological solitons [244,245] and magnetic stripe domains [246,247] are present in 2D magnetic materials. Such structures typically depend on the material’s spin interactions and external-field modulation, showcasing rich interactions and dynamic behaviors in AFM and FM materials [248]. These topological structures in 2D magnetic materials and their topological robustness under extreme conditions offer broad prospects for applications in information storage, spintronics, and quantum computing.

## 7. Conclusions

To sum up, 2D magnetic materials have emerged as a promising avenue for developing next-generation spintronic devices, with their unique properties such as tunable magnetism, spin polarization, and magnetic proximity effects. However, challenges remain in improving these materials’ stability, scalability, and efficiency for industrial applications. Advances in synthesis techniques, such as refined exfoliation and CVD methods, alongside theoretical models like DFT, will be crucial in unlocking the full potential of these 2D magnetic materials. Future work should focus on further understanding the interaction between magnetic domains, enhancing control over spin dynamics, and exploring novel configurations such as magnons, skyrmions, and magnetic moiré superlattices for improved device performances. These efforts will drive significant progress in spintronics, paving the way for more efficient, low-power, and high-speed spintronic applications. Regarding the practical applications of 2D magnets, the following challenges need to be addressed. Firstly, regarding material preparation, 2D magnetic materials currently suffer from issues like low magnetic transition temperatures, poor air stability, and high production costs. While external conditions such as strain, magnetic fields, and electric fields can regulate their magnetic properties, efforts can be made to minimize defects and optimize stacking structures through improved growth techniques. These advancements can improve the material quality; nevertheless, achieving industrial scalability demands will require more universally applicable solutions. Secondly, much remains to be explored in terms of understanding the properties and mechanisms of 2D magnetic materials. Further investigations are needed on magnetic tunneling resistance, magnetic coupling mechanisms, and the material’s tunable magnetic properties to expand their potential applications fully. Lastly, high demands for fast access rates, high sensitivities, and robust stabilities of spintronic applications pose challenges for technically fabricating 2D magnetic materials into functional devices. Controlling spin order in magnetic domains and domain walls in 2D magnets and understanding how external fields influence these magnetic structures will be critical in developing high-performance 2D spintronic devices.

## Figures and Tables

**Table 1:** Summary of magnetic properties for 2D magnetic compounds.

	Material	Magnetic Coupling	Phase Transition, T [K]	Easy Magnetiz-Ation Shaft	Crystal System	Preparation Method	Reference
1. Transition metal halides	CrCl_3_	1L: FM Bulk: AFM	Curie temperature T_C_ = 15 K Neel temperature T_N_ = 17 K	intralayer	monoclinic	mechanical exfoliation CVT	[36,37]
	CrBr_3_	FM	T_C_ = 36 K	interlayer	monoclinic	mechanical exfoliation CVT	[20,38,39]
	CrI_3_	1L: FM 2L: AFM Bulk: AFM	T_C_ = 45 K T_N_ = 61 K	interlayer	monoclinic	mechanical exfoliation CVT	[29,40]
2. Transition metal sulfides	MnO_2_	1L: FM	T_C_ = 140 K		rhombohe-dral	redox reactions	[41,42]
	Cr_2_S_3_	AFM	T_N_ = 120 K (rho-mbohedral) T_N_ = 100 K (trig-onal)		rhombohe-dral/trigo-nal	molecular beam epitaxy CVD	[43,44]
	VSe_2_	FM	Room temperature	intralayer	trigonal	Electrochemical exfoliation method molecular beam epitaxy	[25,45,46]
	Cr_2_Te_3_	FM	T_C_ = 160 K (40.3 nm) T_C_ = 280 K (7.1 nm)	interlayer	hexagonal	CVD	[47]
3. Binary transition metal oxide halides	FeOCl	AFM	T_N_ = 83 K	interlayer	orthorho-mbic	CVT	[48,49]
	CrOCl	AFM	T_N_ = 13.6 K	interlayer	orthorho-mbic	CVT	[50]
	VOCl	AFM	T_N_ = 79 K		orthorho-mbic	CVT	[51]
	CrSBr	intralayer: FM interlayer:AFM	T_N_ = 132 K T_C_ = 146 K	interlayer	monoclinic	CVT CVD	[52]
4. Transition metal compounds	FePS_3_	AFM	T_N_ = 118 K	interlayer	monoclinic	Liquid phase stripping method CVT	[34,53,54]
	NiPS_3_	AFM	T_N_ = 155 K	interlayer	monoclinic	CVT	[55,56,57]
	MnPS_3_	AFM	T_N_ = 80 K	interlayer	monoclinic	mechanical exfoliation	[58,59]
	CoPS_3_	AFM	T_N_ = 120 K	intralayer	monoclinic	CVD	[60]
	CrPS_4_	AFM	T_N_ = 37.9 K	interlayer	monoclinic	CVT	[61]
5. Transition metal germanium tellurium compounds	Fe_3_GeTe_2_	FM	T_C_ = 200 K	interlayer	hexagonal	Al_2_O_3_-assisted mechanical exfoliation three-stage sonication-assisted liquid-phase exfoliation	[32,62]
	Fe_4_GeTe_2_	FM	T_C_ = 270 K	interlayer	rhombohe-dral	CVT mechanical exfoliation	[63,64]
	Fe_5_GeTe_2_	FM	T_C_ = 170 K (1 nm) T_C_ = 240 K (2 nm) T_C_ = 260 K (3 nm)	interlayer		CSCVD	[65]
	Cr_2_Ge_2_Te_6_	FM	2L: T_C_ = 30 K Bulk: T_C_ = 60 K	interlayer	rhombohe-dral	CVD	[66,67]
6. Transition metal bismuth telluride compounds	MnBi_2_Te_4_	AFM	T_N_ = 24 K	interlayer	trigonal	air-quenched methods	[68,69]
	MnBi_4_Te_7_	AFM	T_N_ = 13 K	interlayer	trigonal	flux methods	[70,71]
7. MBene	MnB	FM	T_C_ = 345 K	interlayer	tetragonal	CVD mechanical exfoliation	[72,73]
	CrB	FM	T_C_ = 440 K	intralayer			[74]

## Data Availability

Data are available upon request.
